# Supplementary material for: YEATS2 links histone acetylation to tumorigenesis of non-small cell lung cancer
Source: Nat Commun. 2017 Oct 20;8:1088. doi: 10.1038/s41467-017-01173-4 (PMC5651844; doi:10.1038/s41467-017-01173-4)
Supplement: Supplementary file 1 — Supplementary Information [file 41467_2017_1173_MOESM1_ESM.pdf]

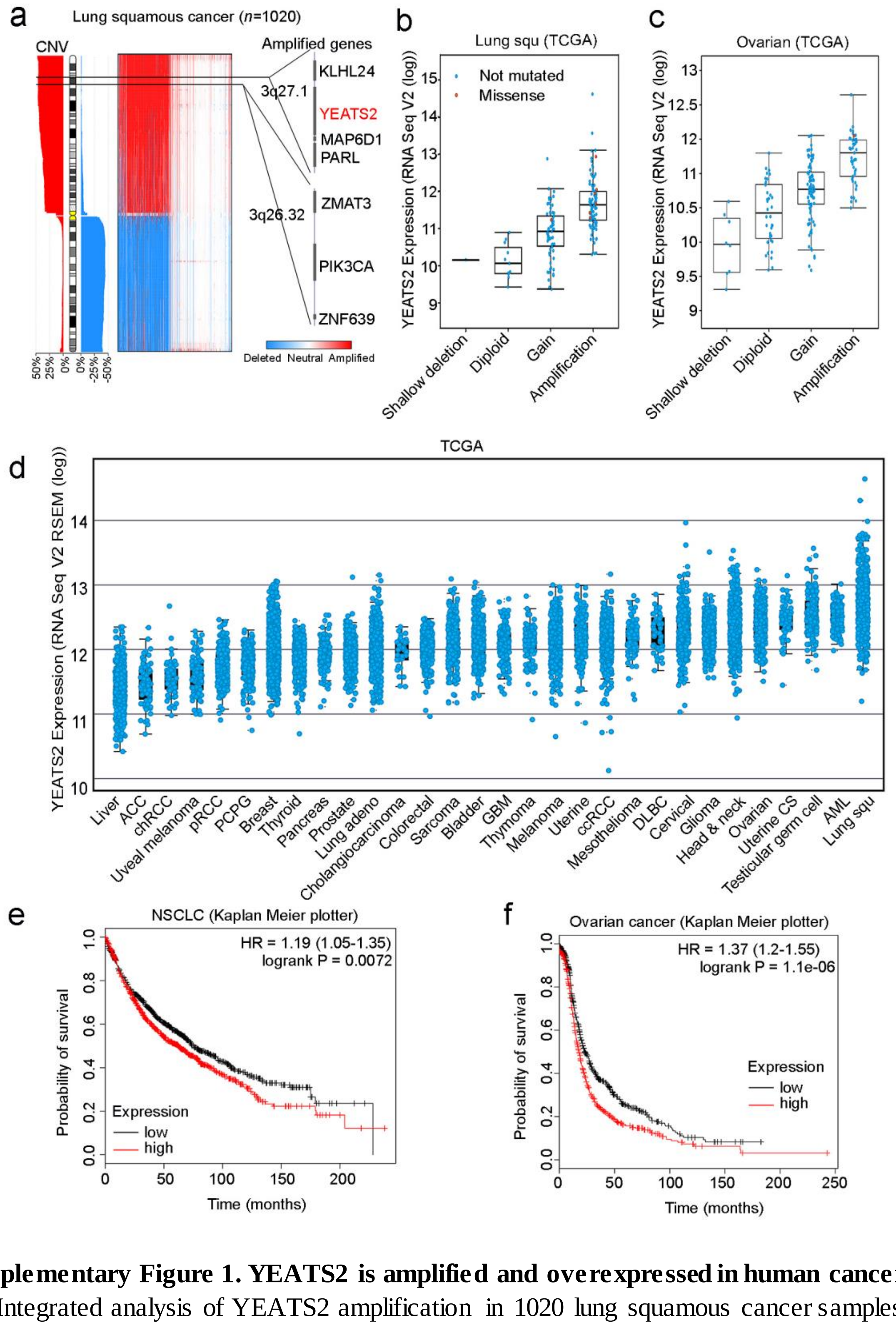

Supplementary Figure 1. YEATS2 is amplified and overexpressed in human cancers.
(**a**) Integrated analysis of YEATS2 amplification in 1020 lung squamous cancer samples from TCGA. Frequency plots of the copy number abnormalities indicate degree of copy-number loss (blue) or gain (red). The color intensity indicates the extent of copy-number changes. Representative genes in the amplicon of chromosome 3q26.32 and 3q27.1 are shown.

(**b**) YEATS2 mRNA levels are positively correlated with gene amplification status in lung squamous cancer. Data were obtained from The Cancer Genome Atlas (TCGA) database.

(**c**) YEATS2 mRNA levels are positively correlated with gene amplification status in ovarian cancer. Data were obtained from The Cancer Genome Atlas (TCGA) database.

(**d**) YEATS2 mRNA levels across different types of cancers. Data were obtained from The Cancer Genome Atlas (TCGA) database.

(**e**) Kaplan-Meier survival curves of NSCLC patients with low or high YEATS2 expression levels. Data were obtained from the Kaplan Meier plotter webserver (http://kmplot.com/). The hazard ratio (HR) was calculated using regression model. *P*-value was calculated using chi-square test.

(**f**) Kaplan-Meier survival curves of ovarian cancer patients with low (black) or high (red) YEATS2 expression levels. Data were obtained from the Kaplan Meier plotter webserver (http://kmplot.com/). The hazard ratio (HR) was calculated using regression model. *P*-value was calculated using chi-square test.

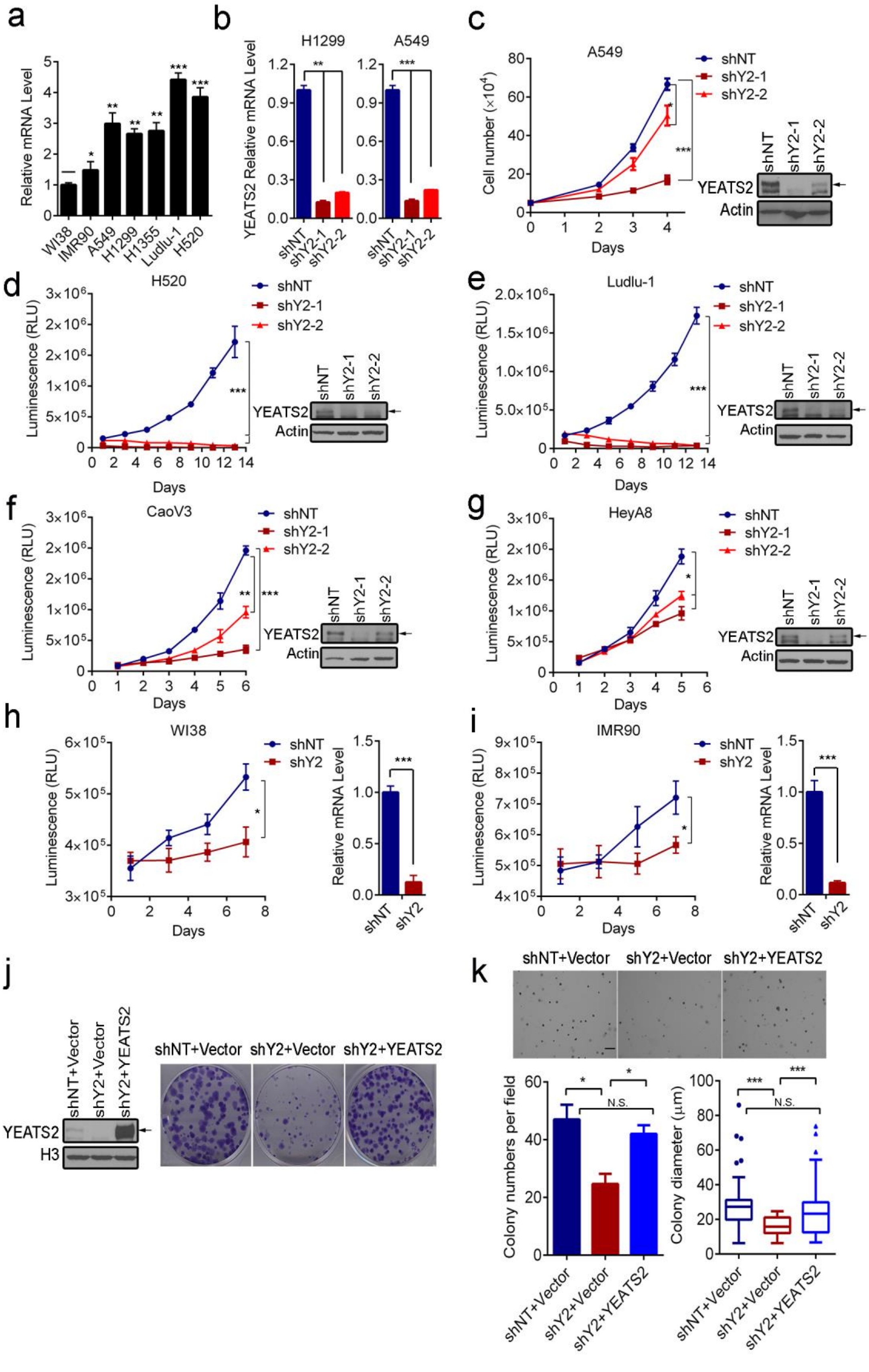
a
Relative mRNA Level
WI38
IMR90
A549
H1299
H1355
Ludlu-1
H520
b
H1299
A549
YEATS2 Relative mRNA Level
shNT
shY2-1
shY2-2
c
A549
Cell number (×10⁴)
Days
shNT
shY2-1
shY2-2
YEATS2
Actin
d
H520
Luminescence (RLU)
Days
e
Ludlu-1
f
CaoV3
g
HeyA8
h
WI38
shNT
shY2
Relative mRNA Level
i
IMR90
j
shNT+Vector
shY2+Vector
shY2+YEATS2
YEATS2
H3
k
shNT+Vector
shY2+Vector
shY2+YEATS2
Colony numbers per field
Colony diameter (μm)
N.S.

Supplementary Figure 2. YEATS2 is required for cell growth and survival.

(**a**) qPCR analysis of YEATS2 mRNA levels in the indicated NSCLC cell lines and immortalized "normal" lung fibroblast cell lines compared to WI38 cell line.

(**b**) qPCR analysis of YEATS2 mRNA levels in control (shNT) and YEATS2 knockdown (shY2) H1299 and A549 cells.

(**c-i**) Cell proliferation assay of NSCLC cells (A549, H520 and Ludlu-1), ovarian cancer cells (CaoV3 and HeyA8), and immortalized lung fibroblast cells (WI38 and IMR90) treated with control (shNT) or YEATS2 shRNAs (shY2). Cell growth (mean ± S.E.M., n=5) was measured 4 days after seeding. Right panels: Western blot analysis showing YEATS2 shRNA knockdown efficiency. The arrow indicates the band of YEATS2 protein.

(**j**) Clonogenic assay of control (shNT) and YEATS2 knockdown (shY2) A549 cells rescued with ectopic expression of YEATS2. Empty vector was used as a control. Colonies were stained and photographed on day 7 after seeding. Left panel: Western blot analysis of YEATS2 expression level in indicated cells. The arrow indicates the band of YEATS2 protein.

(**k**) Anchorage-independent growth assay of cells as in (j). Cells (mean ± S.E.M., n=4-6) were stained with 0.005% crystal violet blue and photographed 3 weeks after seeding. Colony numbers (bottom left) and colony diameters (bottom right) were measured and quantified using ImageJ software. Scale bar, 200 μm.

In all figures, error bars indicate S.E.M. of at least three biological replicates or as indicated. N.S.: not significant, *: $p < 0.05$; **: $p < 0.01$; ***: $p < 0.001$ (Student's t-test).

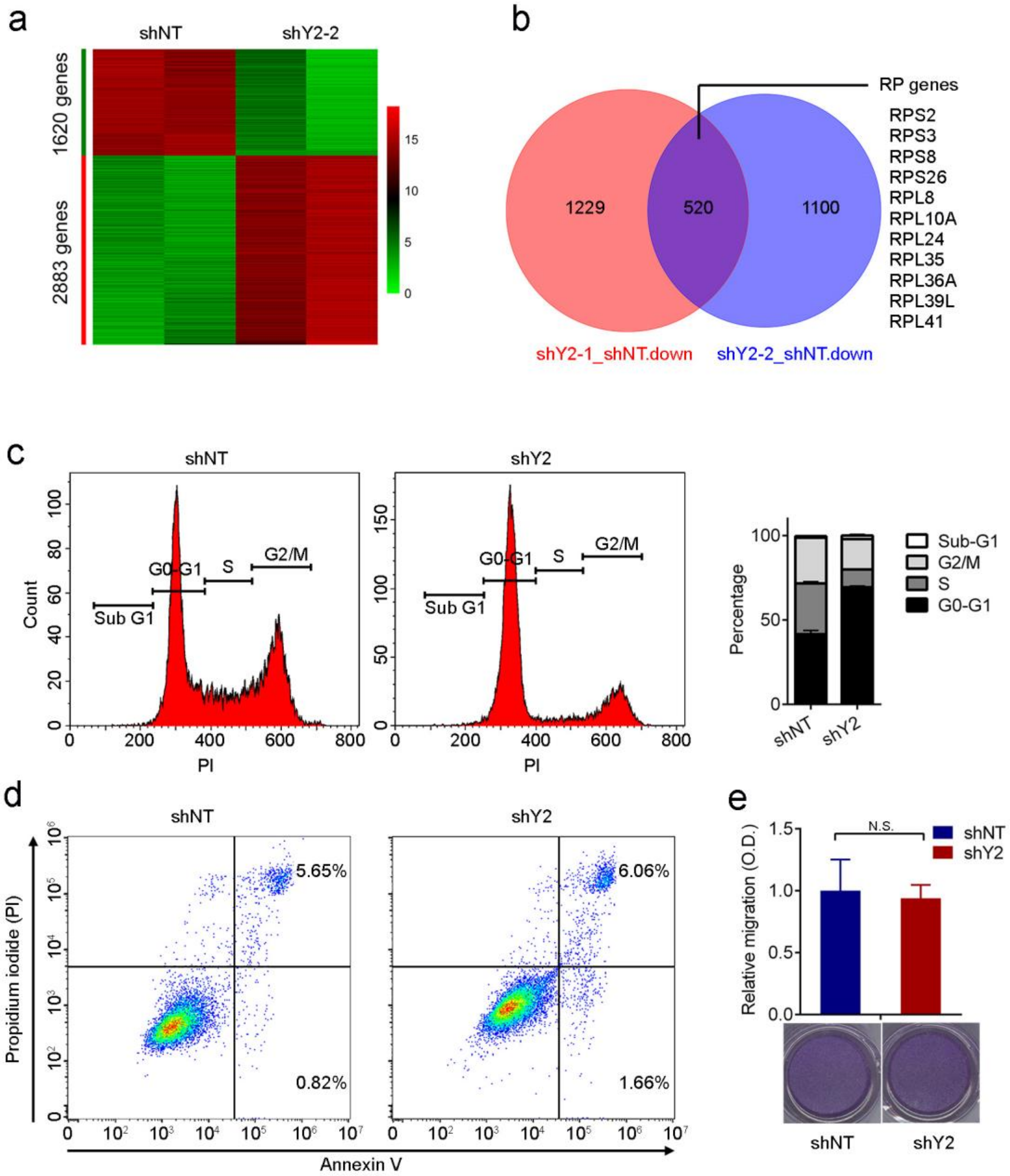

Supplementary Figure 3. YEATS2 KD leads to cell cycle arrest.

(**a**) Heatmap representation of differentially expressed genes in control (shNT) and YEATS2 knockdown (shY2-2) H1299 cells from two independent biological replicates of RNA-seq experiments. Fisher's exact test was used to define differentially expressed genes ($q < 0.01$). The color key represents normalized Log2 expression values.

(**b**) Venn diagram showing the overlap of down-regulated genes in cells treated with two YEATS2 shRNAs (shY2-1 or shY2-2). The overlapped ribosomal protein (RP) genes are listed.

(**c**) Cell cycle analysis of control (shNT) and YEATS2 knockdown (shY2-1) H1299 cells. Cells were stained with propidium iodide and analyzed by flow cytometry to determine the percentage of cells in each stage of the cell cycle. Left panels show representative cell cycle profiling of control cells and YEATS2 KD cells; right panel shows the quantification of cell cycle distribution.

(**d**) Flow cytometry apoptosis analysis of control (shNT) and YEATS2 knockdown (shY2-1) cells. Cells were stained using an FITC Annexin V apoptosis detection kit 4 days after KD. The ratios of the apoptotic cell populations are shown.

(**e**) Trans-well migration assays of control (shNT) and YEATS2 knockdown (shY2-1) cells. Quantifications of three independent experiments are shown in the top panel. N.S.: not significant (Student's t-test).

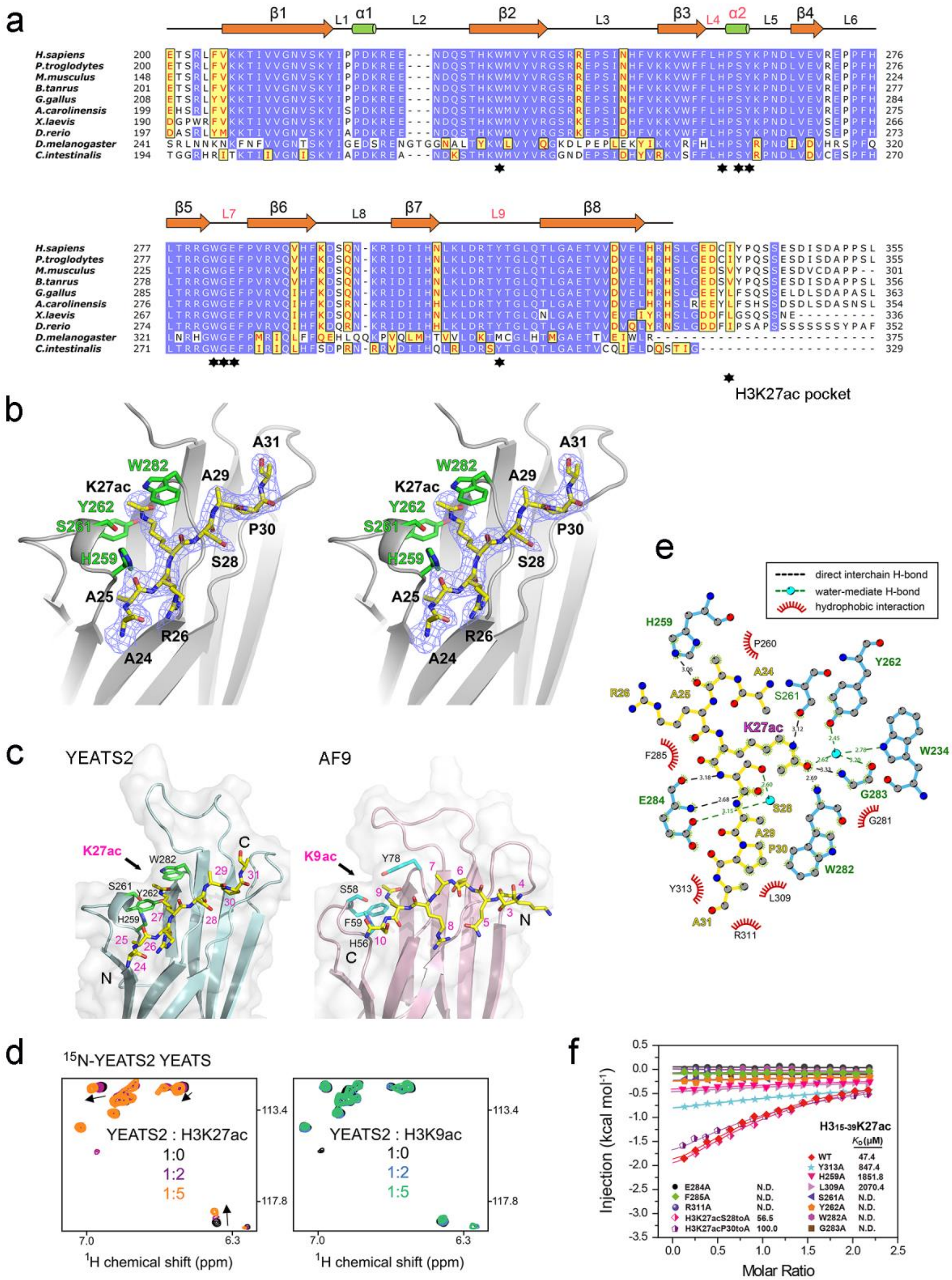

Supplementary Figure 4. The YEATS domain of YEATS2 binds to H3K27ac.

(**a**) Structure-based sequence alignment of YEATS2 orthologs.

(**b**) Stereo electron density map of the YEATS2 YEATS domain around the $H3_{24\text{-}31}K27ac$ peptide. The YEATS domain is shown as grey ribbons, and the histone H3 peptide is depicted as

yellow sticks. Key residues of YEATS2 are depicted as green sticks. Purple mesh, Fo-Fc omit map around $H3_{24-31}K27ac$ peptide contoured at 2.0 σ level.

(**c**) Comparison of the acyl-binding pockets of YEATS2 and AF9. YEATS domains are shown as ribbons, and the histone H3 peptides are depicted as yellow sticks. The amino-terminus (N) and carboxyl-terminus (C) of the H3 peptides are labeled. Left, H3K27ac in YEATS2; right, H3K9ac in AF9 (PDB ID: 4TMP).

(**d**) Superimposed $^{1}H,^{15}N$ HSQC spectra of the YEATS domain of YEATS2 recorded in the presence of increasing concentration of the H3K27ac and H3K9ac peptides. Spectra are color-coded according to the protein: ligand molar ratio.

(**e**) LIGPLOT diagram listing critical contacts between the H3K27ac peptide and the YEATS domain of YEATS2. H3K27ac (yellow) and key residues of YEATS2 (pale blue) are depicted in ball-and-stick mode. Grey ball, carbon; Blue ball, nitrogen; Red ball, oxygen; Cyan ball, water molecule.

(**f**) ITC titration fitting curves of binding of the wild-type YEATS2 YEATS domain or point mutants with the $H3_{15-39}K27ac$ peptide and the wild-type YEATS2 YEATS domain with H3K27acS28A or h3K27acP30A.

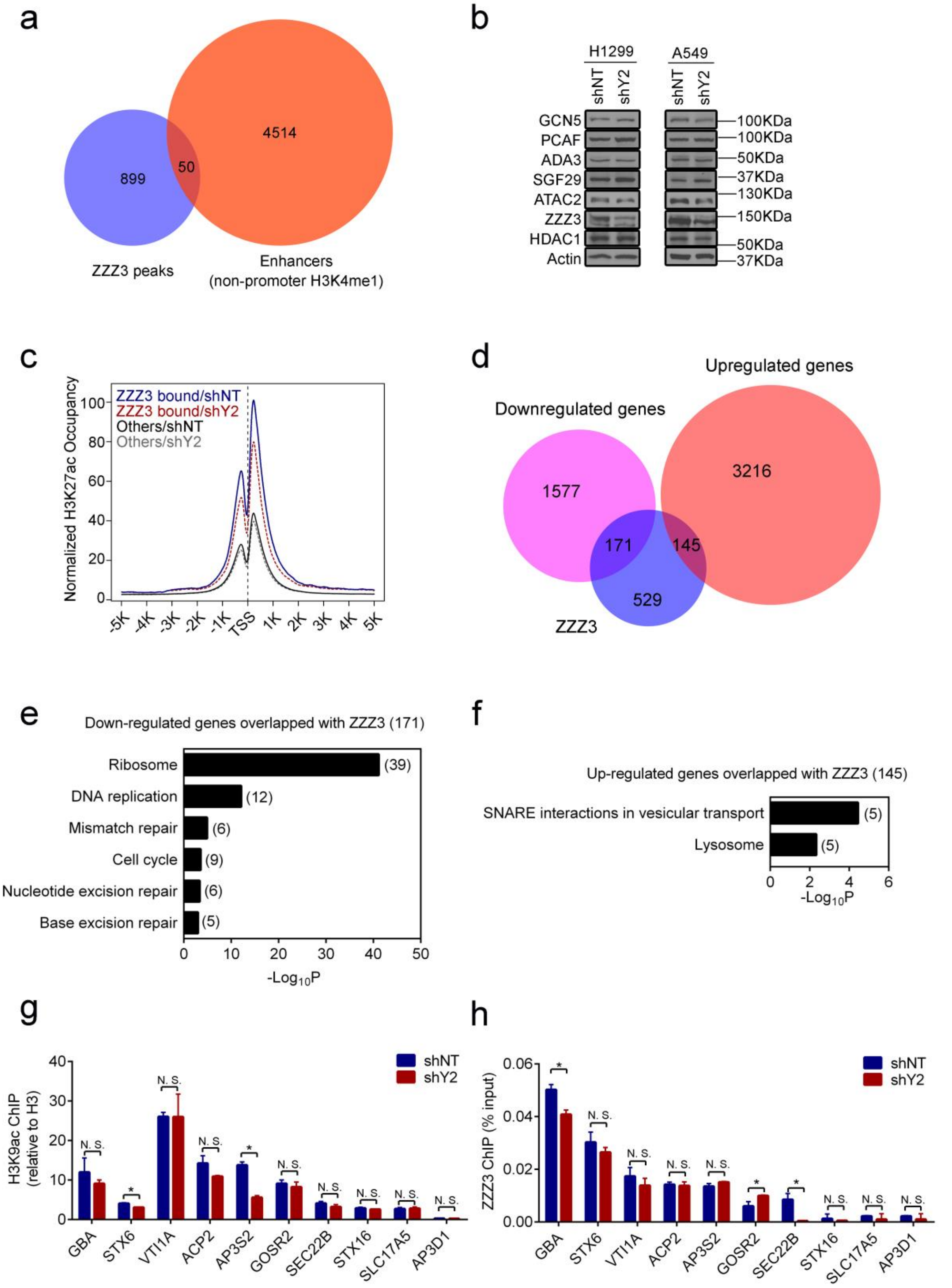

Supplementary Figure 5. The ATAC complex is localized on gene promoters to promote gene activation.

(**a**) ZZZ3 does not localize at enhancers. Vann diagram showing the overlap of ZZZ3 ChIP-seq peaks with enhancer marks (non-promoter H3K4me1 peaks). P = 1 (Super exact test).

(**b**) YEATS2 KD does not affect the ATAC complex protein stability. Western blot analysis of ATAC complex subunits and HDAC1 levels in control (shNT) and YEATS2 KD (shY2) H1299 cells.

(**c**) YEATS2 KD also decreases H3K27ac levels on ZZZ3 bound genes. Average genome-wide H3K27ac occupancy on the promoter (5kb +/- TSS) of the ZZZ3-bound genes or non-ZZZ3 bound genes (others) in control (shNT) and YEATS2 KD (shY2) H1299 cells.

(**d**) Venn diagram showing the overlap of ZZZ3 occupied genes with downregulated or upregulated genes in YEATS2 KD cells.

(**e,f**) KEGG pathway analysis of down- (e) or up-regulated (f) ZZZ3 direct target genes. The numbers of genes within each functional group are shown in parenthesis. Fisher's exact test was used to identify the biological function with significant *P*-values (Benjamini–Hochberg corrected $p < 0.05$).

(**g,h**) YEATS2 KD does not affect H3K9ac levels and ZZZ3 occupancy on most of the upregulated genes in YEATS2 KD cells. qPCR analysis of H3K9ac (g) or ZZZ3 ChIP (h) in control (shNT) and YEATS2 KD (shY2) H1299 cells of the 10 genes enriched in the two pathways of upregulated genes as in (f). Error bars indicate S.E.M. of three biological replicates. N.S.: not significant; *: $p < 0.05$ (Student's t-test).

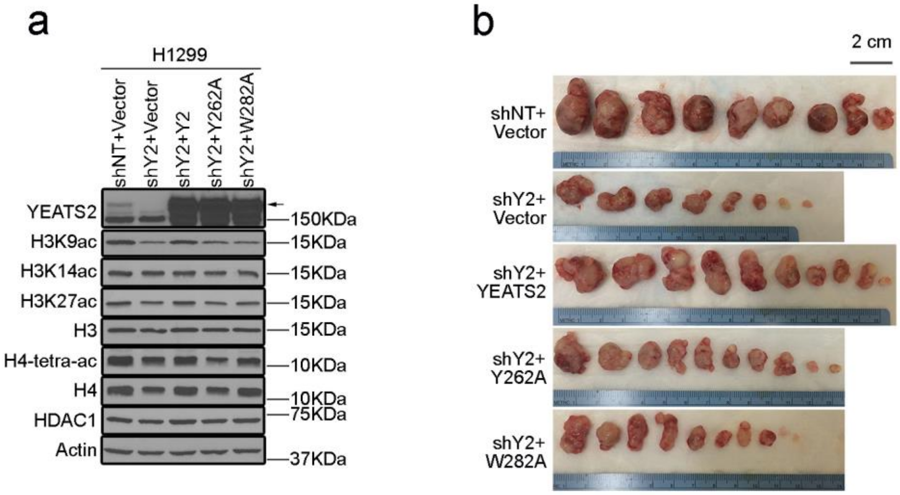

Supplementary Figure 6. The YEATS domain of YEATS2 is required for tumor growth.
(**a**) Western blot analysis of YEATS2, H3K9ac, H3K14ac, H3k27ac, H4-tetra-ac and HDAC1 levels in control (shNT) and YEATS2 KD (shY2) H1299 cells ectopically expressing shRNA-resistant WT YEATS2 or the indicated mutants. The arrow indicates the band of YEATS2 protein.

(**b**) Tumors derived from xenografted H1299 cells as in Fig. 6f. Tumors were collected 5 weeks after subcutaneous transplantation in immunodeficient nude mice.

Fig. 1b

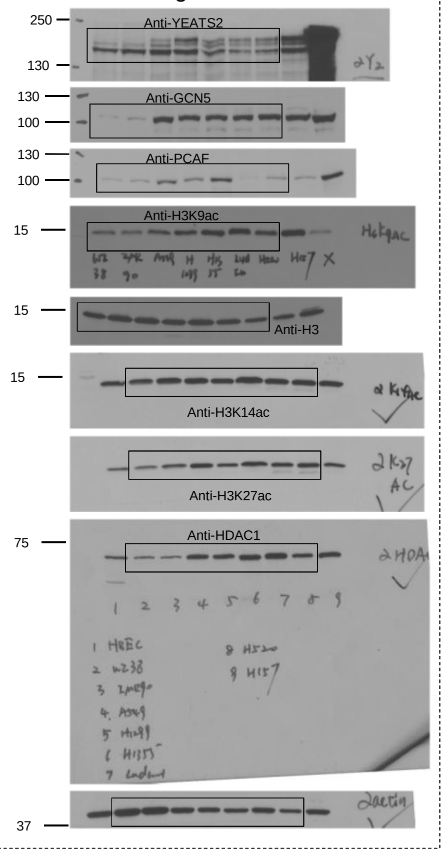

Fig. 3b

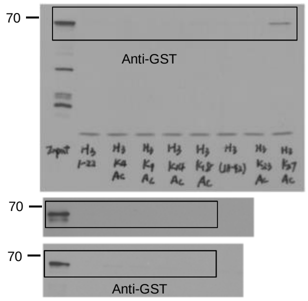

Fig. 3c

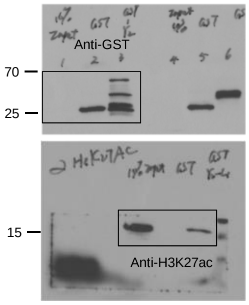

Fig. 3d

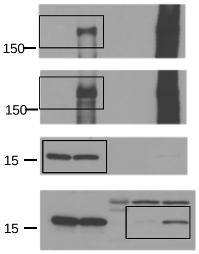

Fig. 3k

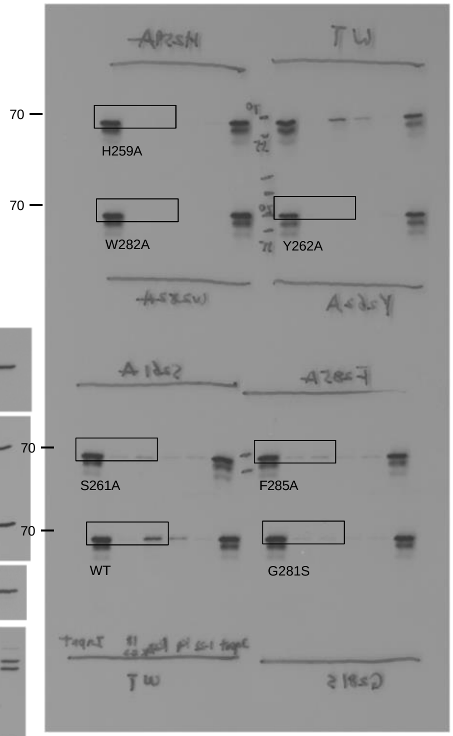

Fig. 5a – H1299

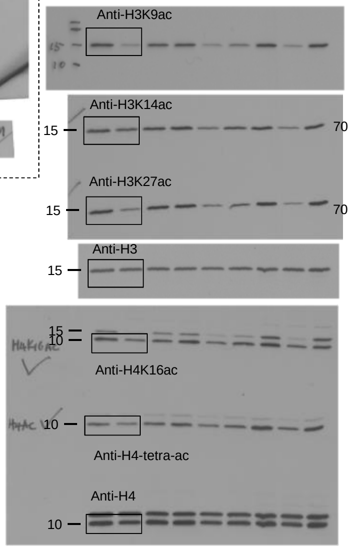

Fig. 1c

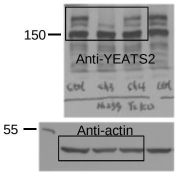

Fig. 1d

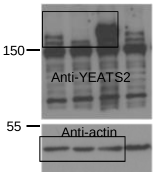

Fig. 5a – A549

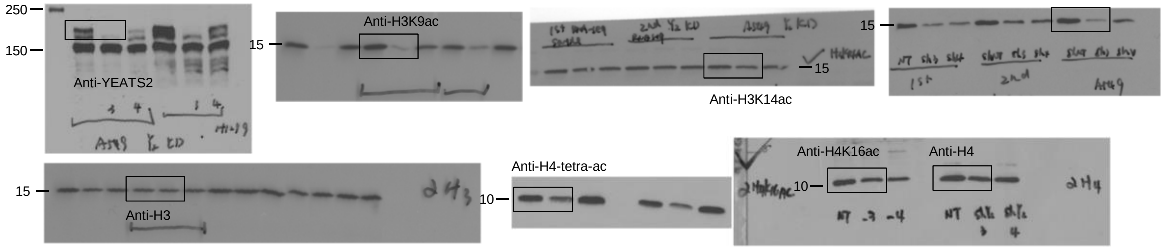

Supplementary Figure 2

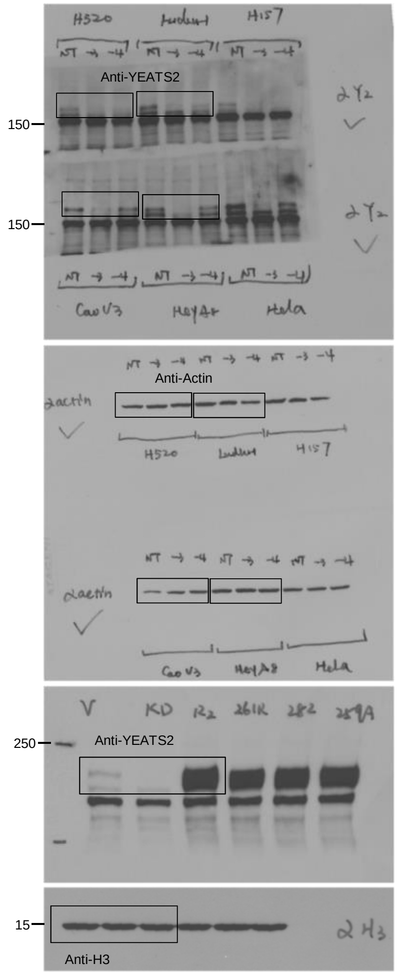

Supplementary Figure 5
H1299

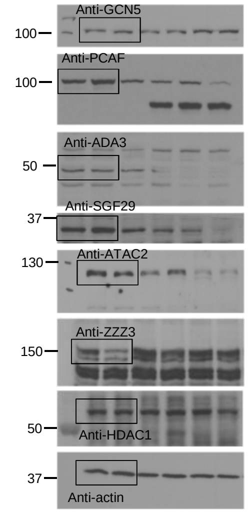

Supplementary Figure 5
A549

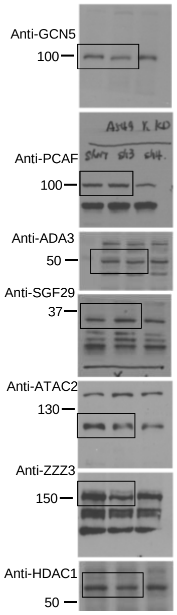

Supplementary Figure 6

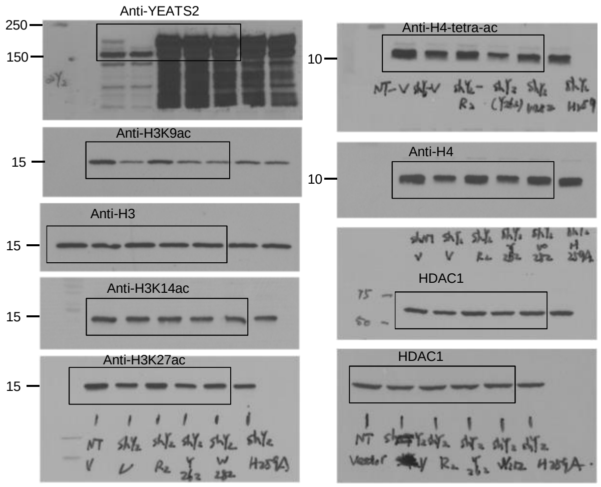

Supplementary Figure 7. Uncropped blots.

Supplementary Table 1. Summary of thermodynamic parameters from ITC

YEATS2 (201-332)	Peptides	ΔH (kcal/mol)	ΔS (cal/mol/deg)	Kd (μM)	N
WT	$H3_{15\text{-}39}$K27ac	-3.08	9.09	47.4	1.0
	$H3_{1\text{-}25}$K18ac	-4.45	2.47	123.3	1.0
	$H3_{1\text{-}34}$K27un	-32.51	-102	4149.4	1.0
	$H3_{6\text{-}15}$K14ac	N.D.			
	$H3_{1\text{-}15}$K9ac	N.D.			
Y313A	$H3_{15\text{-}39}$K27ac	-10.5	-22.3	847.4	1.0
H259A	$H3_{15\text{-}39}$K27ac	-0.03	-11370	1851.8	1.0
L309A	$H3_{15\text{-}39}$K27ac	15.59	0.04	2070.4	0.9
S261A	$H3_{15\text{-}39}$K27ac	N.D.			
Y262A	$H3_{15\text{-}39}$K27ac	N.D.			
W282A	$H3_{15\text{-}39}$K27ac	N.D.			
G283A	$H3_{15\text{-}39}$K27ac	N.D.			
E284A	$H3_{15\text{-}39}$K27ac	N.D.			
R311A	$H3_{15\text{-}39}$K27ac	N.D.			
F285A	$H3_{15\text{-}39}$K27ac	N.D.			
WT	$H3_{15\text{-}39}$ K27acS28toA	-3489	7.33	56.5	1.0
	$H3_{15\text{-}39}$ K27acP30toA	-4029	4.33	100.0	1.0

$H3_{6\text{-}15}$K4ac: TARKSTGGK(ac)A
$H3_{1\text{-}15}$K9ac: ARTKQTARK(ac)STGGKA
$H3_{1\text{-}25}$K18ac: ARTKQTARKSTGGKAPRK(ac)QLATKAA
$H3_{15\text{-}39}$K27ac: APRKQLATKAARK(ac)SAPATGGVKKPH
$H3_{1\text{-}34}$K27un: ARTKQTARKSTGGKAPRKQLATKAARKSAPATGG
$H3_{15\text{-}39}$K27acS28toA: APRKQLATKAARK(ac)AAPATGGVKKPH
$H3_{15\text{-}39}$K27acP30toA: APRKQLATKAARK(ac)SAAATGGVKKPH
N.D.: not detected.

Supplementary Table 2. List of qPCR Primers

Primers	Sequences
YEATS2 RT F	AACCCCAGGGTCTGAATTTATTG
YEATS2 RT R	CGGACGCATACACGTTCCT
RPL6 RT F	CATTACCCCCGGGACCAT
RPL6 RT R	CTTCAGGAAAACCACCCTCTTG
RPL7 RT F	AGTGAGCCCAAAGGTTCGAA
RPL7 RT R	AGCTTCACAAAGGTTCCATTGAA
RPL7A RT F	GGACATCCAGCCCAAAAGAG
RPL7A RT R	TGCCGCTGCAACCTGATATA
RPL8 RT F	GGCCCAGCTCAACATTGG
RPL8 RT R	CCAGGCAGCACACGATTGTA
RPS15 RT F	GAGAAGCCGGAAGTGGTGAA
RPS15 RT R	GTTGAAGGTCTTGCCGTTGTAGA
RPL26 RT F	TGGACAAAGACCGCAAAAAGA
RPL26 RT R	TTTGCCCTTTTCCTTTCCTACTT
RPL27 RT F	ACCTGGGAAGGTGGTGCTT
RPL27 RT R	TTCTTCACGATGACAGCTTTGC
RPL29 RT F	GAACCACACCACACACAACCA
RPL29 RT R	CCCCCTTAAGAGATTCGTATCTTTG
RPL35 RT F	GCGCGTCGCCAAAGTG
RPL35 RT R	ACACGGGCAATGGATTTCC
RPL38 RT F	TGCCTCGGAAAATTGAGGAA
RPL38 RT R	TCTTGACAGATTTGGCATCCTTT
RPL6-ChIP-F	GGCATTCTACCTCACCCTCTTTG
RPL6-ChIP-R	GCCTTCCAGACGCTTCATTT
RPL7-ChIP-F	CGACGGGTTCCACACACAT
RPL7-ChIP-R	AGGACGGAGGTTTTGGAGATC
RPL7A-ChIP-F	GTTCTGATTCCTGCCACTTCACT
RPL7A-ChIP-R	CGGTATTTTAGAGAGGAGAGGATGTG
RPL8-ChIP-F	CCGCGATGCTAACCCTTCT
RPL8-ChIP-R	CCGCCGAGGGCATTC
RPS15-ChIP-F	GCGATGAGGATGCCGATT
RPS15-ChIP-R	GCGTCCTCCTTCCTCTTGAA
RPL26-ChIP-F	AAGAACGGATGGCTGCTGAT
RPL26-ChIP-R	AGCGGGAGCGGGTAAGG
RPL27-ChIP-F	CCAAGGCGACAGTGAACACA
RPL27-ChIP-R	AGCTGACTCCTGCCAGCAA
RPL29-ChIP-F	GCCATCCCCCTCCTAGGA
RPL29-ChIP-R	ACTGGTGACCGACCGTGTGT
RPL35-ChIP-F	CAGGTCGTCCAGCTGTTTCA
RPL35-ChIP-R	GCCAAGATCAAGGCTCGAGAT
RPL38-ChIP-F	TGCAGCCTCGGAAAATTGAG

RPL38-ChIP-R	CACTTACATTTGGCATCCTTTCG
GBA-ChIP-F	ACTTGACCAATGAGACTTGAGGAA
GBA-ChIP-R	GAAAAGAGACGGTCACTCATGC
STX6-ChIP-F	GCAAAAGTATCTCATATGACCAGTGATC
STX6-ChIP-R	CAACCAACAGGACTGCAAAGAG
VTI1A-ChIP-F	CCCTGCGCAGTCTCTGTTACT
VTI1A-ChIP-R	CTTCCGGAGGGAGGTTTGA
ACP2-ChIP-F	CGGAGTCTGCGCTTCGTTA
ACP2-ChIP-R	GCTGGCCTTTGCCCTTTTAG
AP3S2-ChIP-F	AAAATGAACGAAATGTTCCATAACC
AP3S2-ChIP-R	CTTAGCGCTCGCAGAACGT
GOSR2-ChIP-F	CGCCGGCCTCATTTCTC
GOSR2-ChIP-R	ACGCGCGCCTTACTAGCA
SEC22B-ChIP-F	ACCCGCGACCCCAAGT
SEC22B-ChIP-R	CCTAGGAAGGAATCCGATGCT
STX16-ChIP-F	GGTGCGGAAACTGAGTCACA
STX16-ChIP-R	CTACCCCTAACTTTGGCTCGTAAG
SLC17A5-ChIP-F	GGCTCACTTTGCGCCAAT
SLC17A5-ChIP-R	AACTCTGGCTTGGGTTGGACTA
AP3D1-ChIP-F	CGTCAGCTATTACATTCAGGAAAGG
AP3D1-ChIP-R	TGAGCGTGGCTGGATGTG
