# Supplementary material for: YEATS2 links histone acetylation to tumorigenesis of non-small cell lung cancer
Source: Nat Commun. 2017 Oct 20;8:1088. doi: 10.1038/s41467-017-01173-4 (PMC5651844; doi:10.1038/s41467-017-01173-4)
Supplement: Supplementary file 3 — Description of Additional Supplementary Files [file 41467_2017_1173_MOESM3_ESM.pdf]

Description of Additional Supplementary Files

File Name: Supplementary Data 1
Description: Down regulated genes in YEATS2 KD cells

File Name: Supplementary Data 2
Description: Up regulated genes in YEATS2 KD cells

File Name: Supplementary Data 3
Description: GO analysis

File Name: Supplementary Data 4
Description: ZZZ3 ChIP-seq peaks

File Name: Supplementary Data 5
Description: H3K27ac ChIP-seq peaks

File Name: Supplementary Data 6
Description: H3K9ac ChIP-seq peaks

File Name: Supplementary Data 7
Description: ZZZ3 occupied genes
